# Medicines prescribing for homeless persons: analysis of prescription data from specialist homelessness general practices

**DOI:** 10.1007/s11096-022-01399-3

**Published:** 2022-05-23

**Authors:** Aleena Khan, Om Kurmi, Richard Lowrie, Saval Khanal, Vibhu Paudyal

**Affiliations:** 1 School of Pharmacy, College of Medical & Dental Sciences, University of Birmingham, B15 2TT Edgbaston, Birmingham, UK; 2 Faculty of Health and Life Sciences, Coventry University, Coventry, UK; 3 Division of Respirology, Department of Medicine, McMaster University, Hamilton, Canada; 4 NHS Greater Glasgow and Clyde, Glasgow, UK; 5 Division of Health Sciences, Warwick Medical School, University of Warwick, Coventry, UK

**Keywords:** Homelessness, prescribing, inequality

## Abstract

**Background:**

Specialist homelessness practices remain the main primary care access point for many persons experiencing homelessness. Prescribing practices are poorly understood in this population.

**Objective:**

This study aims to investigate prescribing of medicines to homeless persons who present to specialist homelessness primary care practices and compares the data with the general population.

**Setting:**

Analyses of publicly available prescribing and demographics data pertaining to primary care in England.

**Methods:**

Prescribing data from 15 specialist homelessness practices in England were extracted for the period 04/2019-03/2020 and compared with data from (a) general populations, (b) the most deprived populations, and (c) the least deprived populations in England.

**Main outcome measure:**

Prescribing rates, measured as the number of items/1000 population in key disease areas.

**Results:**

Data corresponding to 20,572 homeless persons was included. Marked disparity were observed in regards to prescribing rates of drugs for Central Nervous System disorders. For example, prescribing rates were 83-fold (mean (SD) 1296.7(1447.6) vs. 15.7(9.2) p = 0.033) items), and 12-fold (p = 0.018) higher amongst homeless populations for opioid dependence and psychosis disorders respectively compared to the general populations. Differences with populations in the least deprived populations were even higher. Prescribing medicines for other long-term conditions other than mental health and substance misuse was lower in the homeless than in the general population.

**Conclusions:**

Most of the prescribing activities in the homeless population relate to mental health conditions and substance misuse. It is possible that other long-term conditions that overlap with homelessness are under-diagnosed and under-managed. Wide variations in data across practices needs investigation.

**Supplementary Information:**

The online version contains supplementary material available at 10.1007/s11096-022-01399-3.

## Impact of findings on practice statements

- The majority of prescribing activities focused on homeless populations relates to mental health conditions and substance misuse.
- Our findings suggest potential under-diagnoses and under-treatment of other long-term health conditions such as cardiovascular and respiratory conditions in homeless populations.
- Improving access to prevention and treatment of wider long term health conditions that overlap with homelessness and promoting medicines optimisation and adherence to prescribed treatments are key to improving the health of homeless populations.

## Introduction

An estimated 280,000 people in England are known to be experiencing some form of homelessness [1]. Homelessness includes rooflessness (without a shelter of any kind, sleeping rough); houselessness (with a place to sleep but temporary in institutions or shelter); living in insecure housing (threatened with severe exclusion due to insecure tenancies, eviction, domestic violence); and living in inadequate housing (in caravans on illegal campsites, in unfit housing, in extreme overcrowding) [2]. In addition, in 2019 (i.e. prior to the COVID-19 pandemic), approximately 5,000 people slept rough on any given night in England [1]. During the first phase of the COVID-19 pandemic, an estimated 20,000 households became homeless in England [3]. However, those sleeping rough have been offered temporary accommodations such as emergency shelters and hotels during the pandemic. Findings from published literature suggest that persons experiencing homelessness (PEH) face extreme health inequalities [4] and 12 times higher mortality rates than the general population [5]. In England, males and females PEH are known to die at an average age of approximately 46 years and 43 years, respectively [6]. The majority of higher mortality is contributed by opioid overdose, accidents, heart failures and infectious diseases.

Historically, identifying healthcare and treatment needs amongst PEH has been difficult due to their secluded and unstable locations. Current epidemiological studies on the health status of PEH in the UK have tended to utilise local epidemiological datasets specific to a city, a region or a particular homelessness practice [4, 7]. The emergence of specialist primary care services has enabled accessibility of routine healthcare for PEH across the UK. At least one specialist homelessness general practice or healthcare centre currently operates in most UK cities, providing services exclusively for PEH [8]. Healthcare can also be accessed at general practices with homeless drop-in services, mobile homeless health teams, and volunteer organised healthcare days at local hostels and centres.

Currently, little is understood in regards to the prescribing of medicines to PEH in primary care. Analysis of prescribing data allows understanding of patient access to treatments and enables the development and strengthening of relevant primary care and public health prevention programmes to prevent ill health. One recently published study analysed prescribing medicines to PEH registered in mainstream general practices [9]. Medicines for mental health condition was identified to be the most frequently prescribed items. However, this study only included data of 43 patients registered with mainstream general practices. Two other studies [10, 11]) reported prescribing activities undertaken by pharmacist independent prescribers in specialist homeless health service and outreach settings. Medicines prescribed included treatments for depression, wounds, hypertension, pain and diabetes.

## Aim of the study

This study aims to investigate prescribing medicines for persons who present to specialist general practices for PEH in England and compare the data with general populations.

## Ethics approval

This study undertook an analysis of routinely collected deidentified and aggregated datasets that are publicly available. Therefore, formal ethical approval was not required.

## Method

Prescribing datasets for 12 months between April 2019 and March 2020 relating to 20 British National Formulary (BNF) paragraphs were extracted from 15 specialist general practices for PEH in England through National Health Service (NHS) Digital Openprescribing dataset [12]. Literature mapping specialist primary care services for PEH in England were used to identify locations of specialist practices [8]. Data for specific practices were then searched one at a time in the national primary care prescribing dataset (Openprescribing). Datasets were extracted for each BNF paragraph relating to all general practices in England for comparison with the general populations. In addition, comparisons were made against prescribing datasets from all general practices in the top ten most deprived and ten least deprived Clinical Commissioning Groups (CCGs). The CCGs are clinically-led autonomous NHS bodies involved in providing services for their respective locality and constitute a cluster of general practices within a CCG [13]. The 20 CCGs at either end of deprivation were identified from the Ministry of Housing, Communities & Local Government 2019 summary statistics [14].

The selection of the 20 BNF chapters was based on the list of most commonly prescribed medicines in England in the general populations and epidemiological data related to disease prevalence and Quality and Outcomes Framework (QoF) disease areas [12, 15]. Descriptive analysis included mean and standard deviation of the number of medicinal items prescribed was extracted for mental health (hypnotics and anxiolytics, drugs used in psychoses and related disorders, antidepressant drugs, selective serotonin re-uptake inhibitors, Central Nervous System (CNS) stimulants and drugs used for ADHD, non-opioid analgesics and compound preparations, opioid analgesics, alcohol dependence, nicotine dependence and opioid dependence) and four other major BNF chapters (gastro-intestinal system, cardiovascular system, respiratory system and endocrine system). Populations and demographic data specific to each CCG were extracted from the Office of National Statistics and NHS digital databases [14, 16]. Data were extracted by one author (AK) and checked for accuracy by a second author (VP). Heterogeneity test for prescription items was carried out to compare the data across a) homelessness practices, c) general populations and c) practices in the most deprived and d) least deprived CCGs using a t-test for the continuous variables. All values are reported as the number of items prescribed per 1000 population. All analyses were carried out in Stata version 16, and figures were drawn using a Microsoft Excel sheet.

## Results

The search identified 25 specialist general practices, of which seven practices were not accessible in the *Openprescribing* database, and three services had incomplete datasets and hence were excluded. A total of 15 specialist general practices were included (Table 1) [17–26] in this study, representing 20,572 PEH registrants. Nearly three-quarters of all registrants were males (n = 14,642, 73%).

**Table 1:** Specialist homelessness general practices included in the study

City	Name of practice/ service	Description	Number of Patients
Leeds	York Street Health Practice	Provides primary healthcare services to homeless, vulnerably housed people and asylum seekers [17].	1840
Bradford	Bevan House/ Bevan Healthcare CIC	Specialist primary care organisation providing street medicine services, drop-in centres and soup kitchens for homeless people, refugees, and asylum seekers. Operates Pathway Team assisting patients when discharged from hospital or attending A&E [17].	5439
Coventry	The Anchor Centre	A specialist health centre for homeless and vulnerably housed people. Services include GP clinics, counselling, legal advice, housing advice, community psychiatric nurse weekly clinics, needle exchange, recovery partnership sessions and dental nurse clinics [17].	650
Leicester	Inclusion Healthcare	Provides GP and Nursing clinics for homeless people, residents of hostels, asylum seekers and women working in prostitution over two practices. Additional services include a visiting ophthalmic optician, midwife appointments and a practice therapist for common mental health problems [18].	896
Brighton	Arch Healthcare	Provides a full range of medical service for homeless or temporary housed people, including a drug and alcohol nurse and needle exchange. The practice provides an in-reach service to local hospitals to aid the discharge and follow up care of homeless patients [17].	1602
Southampton	Homeless Healthcare Team	A multi-disciplinary team providing care to homeless, vulnerably housed and refugees. Services also include mental health crisis service and recovery service [19].	526
Oxford	Luther Street Medical Practice	A practice providing a full range of primary care services for homeless and vulnerably housed people. Services include contraception advice, screening for Hepatitis B, C and HIV, assessment/management of mental health, alcohol detoxification, smoking cessation, and leg ulcer management. Additional services include hepatitis B vaccinations, social practitioner assistance, dental, podiatry and psychiatry clinics and acupuncture appointments [20].	487
Chester	St Werburgh’s Practice for the Homeless	Provides diagnostic and screen procedures, the treatment of disease, disorder, or injury for homeless people. It also provides smoking cessation, immunisations, and sexual health services [21].	413
Cambridge	Cambridge Access Surgery	Provides primary health services for homeless people and those at risk of homelessness. The practice also run drug and alcohol clinics twice weekly [17].	669
Exeter	Clock Tower Surgery	Provide healthcare services for homeless and vulnerably housed people. The practice within a wellbeing hub brings together a range of services offering help and support. This includes mental health workers, addiction treatment workers, training, and adult education [22].	570
London, Camden	Camden Health Improvement Practice	A practice that provides primary healthcare for homeless and temporary housed people. Also provides services to people with poor mental health, alcohol, and drug addictions. Services include hepatitis and HIV screening, sexual health screening, contraception, alcohol and drug advice and food vouchers [17, 23].	637
London, Hackney	The Greenhouse Walk-in	Provides healthcare service and housing and welfare advice for homeless people and those at risk of homelessness [17].	1089
London, Newham	Newham Transitional Practice	Delivers comprehensive primary care service to residents who have experienced difficulties registering locally with GP, including homeless people. They provide a special homeless service with several outreach nursing clinics held across Newham [24].	4346
London, Tower Hamlets	Health E1	The practice aims to provide effective and efficient healthcare service to homeless people through daily walk-in clinics. It also offers home visits, family planning, alcohol services, substance misuse and mental health clinics [25].	998
London, Westminster	Great Chapel street medical centre	A flexible service combing medical care and social care by escorting patients to services for specialist interventions. Services included mental health assessment and support, family planning, vaccinations, women’s and men’s health [17, 26].	410

Prescribing data.

Central Nervous system (CNS).

Specialist practices for PEH consistently showed higher rates of prescriptions for the ten CNS drug paragraphs extracted (Table 2). The least deprived regions showed the lowest volume of prescribing. The most commonly prescribed items in the PEH populations were antidepressant drugs, with 4594 items per 1000 population per year. The rate was 3-fold higher than the general populations (p = 0.019) and 3 times higher than the least deprived general populations in England (p = 0.003) (Table 2).

**Table 2:** Number of items prescribed per thousand patients per year (CNS sub-sections). Table represents data from 15 Specialist Homeless General Practices, all general practices in England, General Practices in 10 most deprived CCGs and 10 least deprived CCGs

CNS Sub-section	*Number of Items Prescribed per 1000 Persons*						
	**Specialist General Practices for the Homeless (A)**	**Mainstream (all) General Practices (B)**		**General Practices in Most Deprived CCGs (C)**		**General Practices in Least Deprived CCGs (D)**	
	**Mean (SD)**	**Mean (SD)**	P-Value*	**Mean (SD)**	P-Value**	**Mean (SD)**	P-Value***
Hypnotics and anxiolytics	1513.5 (2806.5)	258.4 (46.8)	0.257	261.6 (108.1)	0.175	204.9 (40.1)	0.157
Drugs used in psychoses and related disorders	2632.4 (2442.7)	218.7 (29.2)	0.018	301.9 (94.0)	0.006	161.4 (33.1)	0.004
Antidepressant drugs	4594.0 (3292.1)	1368.0 (328.5)	0.019	1495.7 (682.1)	0.008	1139.3 (157.9)	0.003
Selective serotonin re-uptake inhibitors	1633.9 (1061.6)	738.5 (167.7)	0.040	786.1 (354.7)	0.024	585.8 (168.1)	0.005
CNS stimulants and drugs used for ADHD	117.6 (182.7)	26.9 (5.0)	0.211	20.4 (11.9)	0.109	25.2(6.3)	0.127
Non-opioid analgesics and compound preparations	773.5 (315.9)	615.4 (165.1)	0.230	806.8 (327.3)	0.801	432.4 (70.1)	0.003
Opioid analgesics	827.2 (957.1)	418.1 (148.5)	0.280	488.8 (266.8)	0.290	304.8 (50.8)	0.100
Alcohol dependence	50.2 (85.6)	2.6 (1.2)	0.161	3.8 (2.5)	0.102	1.2 (0.5)	0.085
Nicotine dependence	114.5 (127.4)	13.0 (3.9)	0.051	12.5 (10.7)	0.020	6.8 (4.1)	0.014
Opioid dependence	1296.7 (1447.6)	15.7 (9.2)	0.033	48.8 (54.6)	0.013	4.0 (4.4)	0.011

* p-value denotes heterogeneity between means of A and B; **p-value denotes heterogeneity between means of A and C; ***p-value denotes heterogeneity between means of A and D

Data concerning drugs related to opioid dependence showed the highest difference in prescribing rates across the PEH and the general population. The rate in the PEH (x = 1296.7 items per 1000 population) was approximately 83-fold higher compared to the general populations (Table 2). Higher prescribing rates were observed for drugs for alcohol dependence (x = 50.2 vs. 2.6 per 1000 population, p = 0.161)) in PEH compared to the general population. Other BNF paragraphs which represented markedly higher prescribing rates in the PEH populations when compared to the general populations were related to drugs used in psychoses and related disorders (x = 2632.4 vs. 218.7 per 1000 population, p = 0.018), selective serotonin re-uptake inhibitors (x = 1633.9 vs. 738.5 per 1000 population, p = 0.040), and nicotine dependence (x = 114.5 vs. 13.0 per 1000 population, p = 0.051). While rates of prescribing opioid analgesics were approximately two-fold higher in the PEH population than the general population, prescribing non-opioid analgesics was similar across the two groups (Table 2).

Cardiovascular system.

Datasets were extracted for beta-blockers, calcium channel blockers (CCB), renin-angiotensin-aldosterone system (RAAS), antiplatelets and lipid-regulating drugs (Table 3, Electronic supplement 1). Fewer items were prescribed of RAAS drugs (x = 760.5 vs. 1186.3 per 1000 population, p = 0.005), antiplatelet drugs (x = 442.7 vs. 599.2 per 1000 population, p = 0.085), lipid-regulating drugs (x = 1043.9 vs. 1378.8 per 1000 population, p = 0.086) in PEH population compared to the general populations. The rates of prescribing these drugs in PEH populations were even lower when compared to the most deprived populations in England (Table 3, Electronic supplement 1).

**Table 3:** Number of items prescribed per thousand patients per year (Non-CNS chapters). Table represents data from 15 Specialist Homeless General Practices, all general practices in England, General Practices in 10 most deprived CCGs and 10 least deprived CCGs

BNF Chapters	Sub-sections	*Number of Items Prescribed per 1000 Persons*						
		**Specialist Homeless General Practices (A)**	**Mainstream (all) General Practices (B)**		**General Practices in Most Deprived CCGs (C)**		**General Practices in Least Deprived CCGs (D)**	
		**Mean (SD)**	**Mean (SD)**	P-Value*	**Mean (SD)**	P-Value**	**Mean (SD)**	P-Value***
**Gastro-Intestinal System**	Proton Pump Inhibitors	1870.2 (1151.4)	1145.5(236.6)	0.119	1234.8(468.6)	0.113	929.1(121.8)	0.018
**Cardiovascular System**	Beta-Adrenoceptor Blocking Drugs	774.4 (483.4)	717.8(176.3)	0.769	772.8 (340.3)	0.993	591.3(70.5)	0.250
	Renin-Angiotensin Drugs	760.5 (333.6)	1186.3(177.9)	0.005	1262.9(429.5)	0.003	1065.4(81.3)	0.010
	Calcium-Channel Blockers	754.1 (726.6)	769.6(99.2)	0.956	862.1(241.7)	0.656	672.2(54.1)	0.727
	Antiplatelet Drugs	442.7 (213.1)	599.2(113.4)	0.085	679.6(244.5)	0.017	467.5(54.8)	0.724
	Lipid-Regulating Drugs	1043.9 (459.8)	1378.8(234.6)	0.086	1562.2(450.5)	0.011	1111.0(118.5)	0.658
**Respiratory System**	Corticosteroids (respiratory)	487.7 (228.8)	393.0(81.0)	0.305	452.8(154.9)	0.678	330.2(35.8)	0.042
**Infections**	Penicillins	313.1 (116.0)	267.3(25.4)	0.319	295.9(60.0)	0.670	230.5(22.1)	0.037
**Endocrine System**	Antidiabetic drugs	710.8 (447.2)	749.2(96.5)	0.826	1185.1(1119.9)	0.151	761.6(379.8)	0.771
	Thyroid hormones	191.3 (157.5)	602.2(101.9)	< 0.001	546.6(206.9)	< 0.001	548.4(73.3)	< 0.001

* p-value denotes heterogeneity between means of A and B; **p-value denotes heterogeneity between means of A and C; ***p-value denotes heterogeneity between means of A and D

Gastro-Intestinal system.

Prescribing data for proton pump inhibitors (PPIs) were extracted (Table 3, Electronic supplement 1). Significantly more items per 1000 population were prescribed to the PEH population than the general population (x = 1870.2 vs. 1145.5 per 1000 populations, p = 0.019).

Respiratory system.

Respiratory corticosteroids datasets were extracted (Fig. 1). Prescriptions in specialist practices for the homeless were approximately 20% higher than in mainstream practices (Table 3, Electronic supplement 1).

Endocrine system.

Datasets for antidiabetic drugs and thyroid hormones were extracted (Fig. 1). Fewer rates of antidiabetic medicines (x = 710.8 vs. 749.2 p = 0.826) and thyroid hormones (x = 191.3 vs. 602.2, p < 0.001) were prescribed in the PEH populations when compared to the general populations (Table 3, Electronic supplement 1).

Infections.

The prescribing frequency of anti-infectives was higher in the PEH population than mainstream general practices (x = 313.1 vs. 267.3 p = 0.319) and compared to the practices in the most and least deprived areas of England (Table 3, Electronic supplement 1).

## Discussion

### Statement of key findings

This study aimed to investigate trends of medicine prescribing to PEH populations in England. Datasets from 15 specialist general practices were retrieved and analysed, representing over 20,000 patients. Results demonstrate significantly higher prescribing rates for substance misuse and mental health conditions in the PEH than in the general population. However, fewer items aimed at other long-term health conditions, including cardiovascular and endocrine conditions, were prescribed to PEH compared to the general populations, suggesting that long-term conditions amongst PEH may be underdiagnosed and/or undertreated. Undiagnosed and under-treated long-term health conditions could explain the early and higher mortality rates observed in the PEH populations [6].

There were large variations in the rates of prescribing across the specialist homelessness general practices. Differences were most notably identified for mental health conditions, where some specialist general practices prescribed specific medicines categories approximately ten times more items than other specialist practices. Local commissioning arrangements in the services, discrepancies in local guidelines and formularies such as prescription thresholds and referral to mental health teams may have influenced such variations.

### Strengths and weaknesses

The datasets represent a large sample of PEH from over 15 specialist general practices located in major urban areas across England, and the general population dataset provided a robust comparison. Given the large population size and completeness of Openprescribing dataset [12], any possibility of missing data is likely to be small and balanced across the comparison groups. Prescribing data in this study relied on registrants accessing healthcare exclusively from their specialist primary organisation. It is important to note that not all PEH have access to specialist homelessness health services, and some PEH may also be receiving services in mainstream general practices. However, previous studies have demonstrated PEH exclusion from mainstream services due to complexity in navigating services, inability to keep up appointments and perceived stigma and discrimination in practices by other patients and healthcare professionals [27–29]. These factors lead to PEH preferring to use specialist practices for their health services use. PEH are also frequent users of emergency departments and may often be excluded altogether from primary care [30]. Prescribing data was collected at a practice level, and therefore due to lack of access to patient-level data, it is important to undertake careful consideration. Large variations in prescribing rates were observed amongst specialist practices demonstrating the impact of local policies, practice level demographics, small sample size of the practices and unknown factors.

### Interpretation

Epidemiological studies have demonstrated a high prevalence of mental health conditions and substance misuse problems [4, 7]. Causal and consequential relationships between mental health conditions, homelessness and substance misuse have been established [31, 32]. A recent systematic review showed that PEH are more likely to have a 3-fold higher risk of cardiovascular disease (OR 2.96) and 138% higher hypertension (OR 1.38) than general populations [33]. Literature suggests approximately 70% higher mortality rates in PEH due to all cardiovascular-related causes compared to the general population [34, 35]. Prescribing rates of cardiovascular drugs was observed to be lower in specialist homelessness practices in the current study compared to the general population suggesting potential under-diagnosis and under-treatment. Factors contributing to under-diagnoses and treatment can include prioritising treatment of substance misuse and mental health from patients and prescriber perspectives, asymptomatic nature of other long term health conditions, lack of holistic screening of health conditions and time constraints. In addition, long-term health and mortality compete with more immediate needs, such as obtaining adequate food and shelter. However, the literature suggests that PEH are willing to accept healthcare for chronic conditions if given the opportunity, and they believe such care to be important [36]. In relation to acute conditions, higher prescribing rates were observed with antibiotics demonstrating their vulnerability to injuries, infections and lack of opportunity to undertake self-care [37]. In addition, poor nutrition and stress increase the risk of exposure to a range of respiratory tract infections, viruses, and diseases.

### Further research

Higher rates of prescribing of medicines for substance misuse and mental health demonstrate patient and/or prescriber priorities about their healthcare needs. Optimisation of prescribed medicines is important to ensure optimal outcomes from their treatment [38]. Introduction of prescribing pharmacists and nurses and outreach-based services conducted by the primary care team are likely to promote medicines optimisation and engagement with care [39, 40]. There is a need to develop and evaluate innovative service models to encourage access to services and treatments. Innovative service models are important also in the light of the recent COVID-19 pandemic, which has led to disruption in routine care provision [41–43] and is likely to widen inequality in access to service provisions. While our study demonstrated high prescribing rates in substance misuse and mental health, promoting adherence to prescribed medicines is key to optimal treatment outcomes. Patients with a dual diagnosis of mental health and substance misuse are most vulnerable to facing homelessness [44], and hence primary health and social care services should offer tailored services to prevent key causes and consequences of homelessness.

Many PEH are excluded from primary care due to various factors such as lack of ability to navigate services, perceived stigma and discrimination in healthcare settings and wrong application of registration criteria in mainstream practices [17]. It is imperative to capture the health status of those who do not have access to any primary care services. This study used aggregated dataset at practice level and future studies should consider exploring the extent of disparity using patient level data.

## Conclusions

Currently, most of the prescribing activities in PEH relates to mental health conditions and substance misuse. It is possible that other long-term health conditions, such as diabetes and cardiovascular diseases, may be under-diagnosed and undermanaged. Primary healthcare professionals should offer targeted and opportunistic screening and treatment for other health conditions that overlap with poverty and deprivation. Optimisation of prescribed treatments and promoting adherence is key to achieving favourable health outcomes.

Data statement: Openprescribing datasets are developed and maintained by the University of Oxford EM DataLab [12]. It claims to cover anonymised data about the drugs prescribed by all general practices in England. However, it is likely that some general practices may be missing in the databases, particularly the specialist homelessness practices as reported in the results section of this paper. Data are shown per 1000 populations and are adjusted for England’s mid-year population size every year. The data are also normalised by drug name and classification and therefore, when drugs change their name or move across BNF chapters, it does not interfere with the analysis [45].

## Electronic Supplementary Material

Below is the link to the electronic supplementary material.

Supplementary Material 1

## Data Availability

All data generated or analysed during this study are included in this published article (and the supplemental materials).
