# Supplementary material for: Medicines prescribing for homeless persons: analysis of prescription data from specialist homelessness general practices
Source: Int J Clin Pharm. 2022 May 23;44(3):717–24. doi: 10.1007/s11096-022-01399-3 (PMC9126241; doi:10.1007/s11096-022-01399-3)

Electronic supplement 1: Mean number of items prescribed/1000 patients/year for all practice types. General Population (Mainstream General Practices), Low IMD rank (General Practices in Most Deprived CCGs) and High IMD rank (General Practices in Least Deprived CCGs).


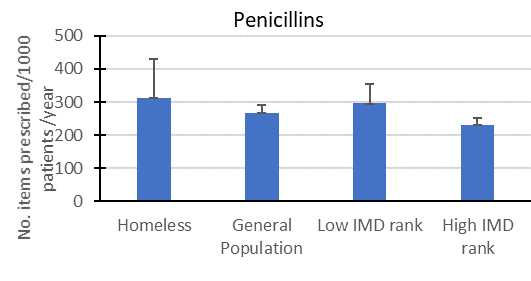

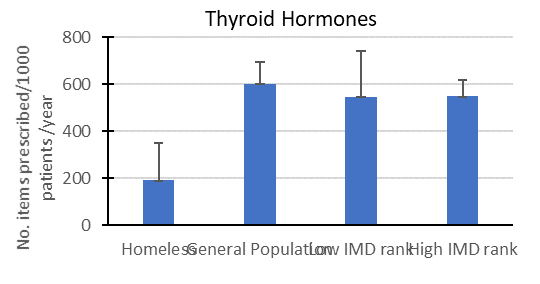

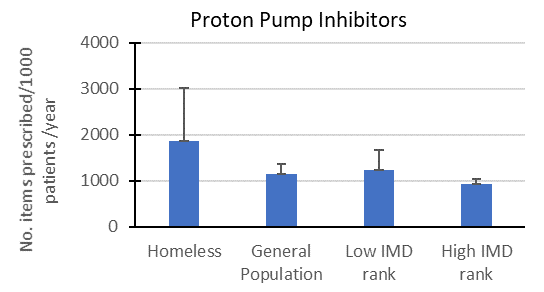

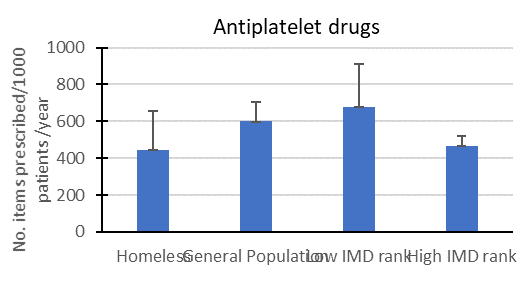

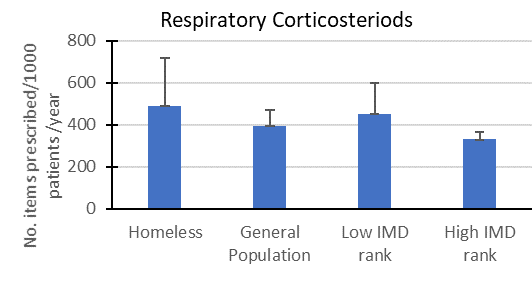

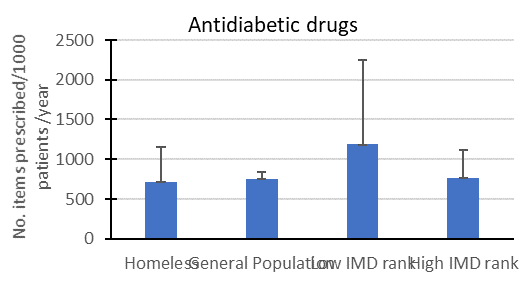

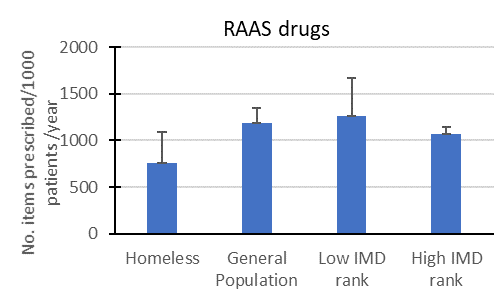

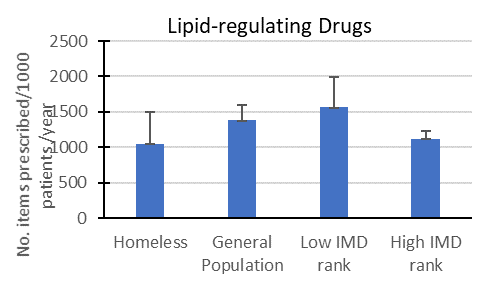

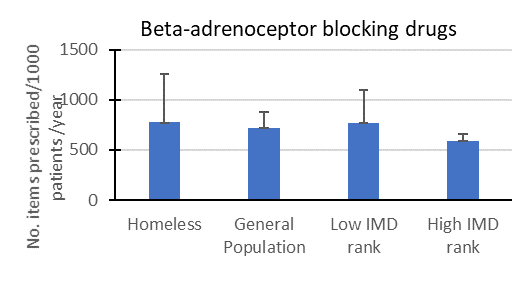

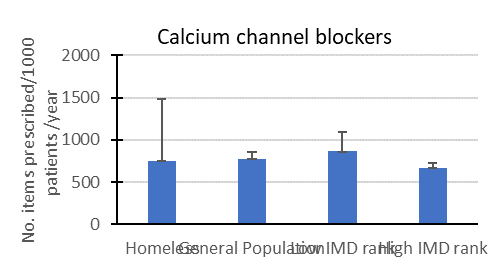

Supplement: Supplementary file 1 — Supplementary Material 1 [file 11096_2022_1399_MOESM1_ESM.docx]
